# A quantitative amplitude of vagus nerve obtained by intraoperative neuromonitoring predicts postoperative vocal cord paralysis among patients in thyroid/parathyroid surgery as a second option

**DOI:** 10.1016/j.sopen.2024.12.009

**Published:** 2025-01-04

**Authors:** Hiroshi Katoh, Riku Okamoto, Kanako Naito, Tomoya Mitsuma, Mariko Kikuchi, Takaaki Tokito, Takeshi Naitoh, Naoki Hiki, Yusuke Kumamoto, Takafumi Sangai

**Affiliations:** a Department of Breast and Thyroid Surgery, Kitasato University Hospital/Kitasato University School of Medicine, Kanagawa, Japan; b Department of Lower Gastrointestinal Surgery, Kitasato University Hospital/Kitasato University School of Medicine, Kanagawa, Japan; c Department of Upper Gastrointestinal Surgery, Kitasato University Hospital/Kitasato University School of Medicine, Kanagawa, Japan; d Department of General-Pediatric-Hepatobiliary Pancreatic Surgery, Kitasato University Hospital/Kitasato University School of Medicine, Kanagawa, Japan

**Keywords:** Intraoperative nerve monitoring, Vocal cord paralysis, Vagus nerve, Quantitative amplitude, Thyroid disease, Parathyroid disease

## Abstract

**Background:**

The advantage of intraoperative neuromonitoring (IONM) has been widely accepted in thyroid/parathyroid surgery. However, there are discrepancies of amplitudes on recurrent laryngeal nerve (RLN) palsy and vocal cord paralysis (VCP) because of amplitude variations among individuals. Accordingly, the universal usefulness of quantitative amplitude value *per se* among patients were assessed.

**Study design:**

IONM using a 4-step method (Vagus nerve (V1)-RLN (*R*1)-R2-V2) was applied to 777 RLNs (510 patients). Forty-nine RLNs were excluded because of either loss of signal without preoperative VCP or combined RLN resection. The remaining 728 RLNs were evaluated. The optimal cut-offs of amplitudes or ratios of amplitude decrease on VCP were determined and evaluated. An independent recent cohort (177 RLNs) was analyzed for validation.

**Results:**

Quantitative amplitudes of V2 or R2, and V2/V1 or R2/R1 ratio predicted VCP. The V2 of 117–216 μV predicted VCP with high (>80 %) sensitivity and specificity. Interestingly, the AUC of ROC curve of V2 was the highest, and a cut-off 124 μV of V2 most excellently predicted VCP with the highest sensitivity, specificity, and both positive and negative predictive values. In dissociative analyses, a V2 cut-off 124 μV still excellently predicted VCP in all ranges of initial V1 ≥ 100 μV. In a validation cohort, the V2 of 126–205 μV (cut-off 197 μV) predicted VCP with both high (>80 %) sensitivity and specificity.

**Conclusions:**

A quantitative V2 amplitude can predict postoperative VCP among individuals as a simple and a second option, that may be especially useful in some circumstances with unavoidable insufficient initial exposure of vagus nerve.

## Introduction

Dysfunction of recurrent laryngeal nerve (RLN) palsy and vocal cord paralysis (VCP) is the most common complication and the one of most priority to be protected in thyroid and parathyroid surgery. The advantage of intraoperative neuromonitoring (IONM) for identification of RLN has been widely accepted and *de facto* standard in high-risk patients such as large goiter, substernal goiter, Graves' disease, and central neck dissection for thyroid cancer, contributing to protection of RLN function [[1], [2], [3], [4], [5], [6], [7]]. An electromyogram amplitude represents twitch of posterior cricoarytenoid muscle or cricothyroid muscle, reflecting integrity of RLN or external branch of superior laryngeal nerve, respectively. The International Neural Monitoring Study Group (INMSG) guideline describes the standard methods for improvement of quality of IONM [2,3]. A remarkable decrease or loss of the electromyogram (EMG) amplitude of IONM implies RLN palsy and VCP during surgical procedure. However, there are many discrepancies of IONM amplitudes for prediction of postoperative VCP and nonnegligible variation of amplitudes among individuals. And a relative decrease of amplitude of IONM (*i.e.* remarkable decrease or loss of an amplitude) is main usage to evaluate RLN integrity in each patient and the usage of amplitude values is only limited to an individual. Accordingly, the clinical significance of actual quantitative amplitude *per se* obtained by IONM is still elusive. Besides, we often experience insufficient initial amplitudes (<500 μV) even after tube repositioning with superficial electrodes regardless of the guideline recommendation, and unavoidable insufficient initial exposure of vagus nerve in some circumstances such as tumor extension and adhesive operation field. Therefore, the aim of this study is to evaluate that the diagnostic accuracy of a quantitative amplitude value and whether a quantitative amplitude value *per se* can be applicable universally among patients to predict RLN integrity and postoperative VCP. Here, we propose simplified universal application of a quantitative IONM amplitude of vagus nerve stimulation at the end of surgical procedure as a second option.

## Patients and methods

Among 1784 patients who underwent surgery for thyroid/parathyroid diseases at Kitasato University Hospital from March 2015 to September 2023, we retrospectively evaluated 777 recurrent laryngeal nerves (RLNs) at risk in 510 patients (28.6 %) (519 diseases) in whom quantitative intermittent (I)-IONM were applied. Nine patients had multiple diseases. Taking the balance between the cost and benefits, a quantitative I-IONM using a mechanically stimulated EMG response on the monitor was preferentially conducted in a high-risk patients (*eg.* large goiter, substernal goiter, extrathyroidal extension of cancer, or previous surgical history of paratracheal area) according to a surgeons' discretion. A manual twitch detection of posterior cricoarytenoid muscle was performed in the remaining patients in whom a quantitative IONM using EMG response was not applied during the study period. Laryngeal fiber scope was conducted to objectively determine VCP and RLN palsy in all patients within 1 month prior to surgery, 2 or 3 days after surgery. In case of postoperative VCP, laryngeal fiber scope was performed 1, 3, 6, and 12 months after surgery until recovery from VCP. After 1 year from surgery, laryngeal fiber scope was conducted every 1 year until 3 years after surgery for patients with VCP and related symptoms such as dysphagia and aspiration. Clinicopathological information was collected from the electronic medical records of Kitasato University Hospital. An independent recent cohort of 177 RLNs at risk in 140 patients who underwent surgery without combined resection of RLN were also analyzed for validation.

This study was approved by the ethics committee (IRB) of the Kitasato University School of Medicine (the IRB approved #22-004), and was performed in accordance with the clinical research guidelines of the IRB of the Kitasato University School of Medicine. The approach of opt-out consent was employed for this retrospective analysis. The study was conducted in accordance with the Declaration of Helsinki (as revised in 2013).

### Intraoperative nerve monitoring (IONM)

Anesthetic induction was achieved with total intravenous anesthesia (TIVA) by propofol and remifentanil combined with standard dose rocuronium (0.6 mg/kg) in all patients. Intubation was performed using a Standard reinforced EMG tube (Medtronic) in 5 patients, a TriVantge EMG tube (Medtronic) in 511 patients, or a Laryngeal Electrode (Inomed) in 4 patients using a McGRATH (Medtronic). Initial confirmation of electrodes positioning was done by tap test. Anesthetic maintenance was achieved with TIVA. When the initial amplitude of either vagus nerve (V1) or RLN (R1) was <200 μV under 1 mA stimulation (3 mA stimulation in endoscopic thyroidectomy/parathyroidectomy), the tracheal tube was repositioned under direct laryngoscopy. A reversal agent (sugamadex) was used when initial baseline amplitude of either V1 or R1 was still <200 μV (116 patients, 22.7 %). Intermittent (I)-IONM was performed using the following neuromonitoring systems: a NIM-response 2.0 (68 patients), a NIM-response 3.0 (438 patinets) (Medtronic), or C2 Xplore (Inomed) (4 patients), in combination with continuous (C)-IONM with APS electrode (Medtronic) (15 patients) or Delta electrode (Inomed) (1 patient).

General 4-step method was used for I-IONM following the 2011 INMSG guideline and the previous literatures [[2], [3], [4],[6], [7], [8]]. Briefly, a vagus nerve and a RLN were stimulated at first exposure as V1 and R1, and before wound closure as V2 and R2 at the most proximal exposed part. An amplitude under 1 mA stimulation of both vagus nerve (V1, V2) and RLN (R1, R2) was used for this study except an amplitude of vagus nerve in total endoscopic thyroidectomy/parathyroidectomy where vagus nerve was stimulated roughly *via* carotid sheath under 3 mA to obtain sufficient amplitudes. Intraoperative loss of the neuromonitoring signal (LOS) was defined as decrease of the nerve amplitude to less than half of a baseline amplitude. LOS was subdivided to type 1 and type 2 [6]. Type 1 was diagnosed with segmental LOS distal side of a specific focal point of the exposed RLN. Type 2 was diagnosed with global LOS of the exposed RLN. According to the subtype of LOS throughout operation, postoperative VCP was classified to Type 1, Type 2, and unknown (without LOS during operation).

### Statistical analysis

A chi-squared test or a Fisher's exact test was applied for correlation analysis of categorical variable, where appropriate. A Student's *t-*test or a Mann-Whitney *U* test was used for comparison of continuous variables of two groups, where appropriate. The follow-up time was calculated from the date of surgery. For an amplitude obtained from IONM, an ROC curve was used to determine an optimal cut-off value on postoperative VCP using a Youden's index. The cut-off of value was used to classify patients to two groups to predict postoperative VCP. A *p* < .05 was considered significant. All statistical analyses were conducted using a JMP Pro17 (SAS Institute).

## Results

### Patient characteristics and main reasons for IONM application

**Fig. 1:**
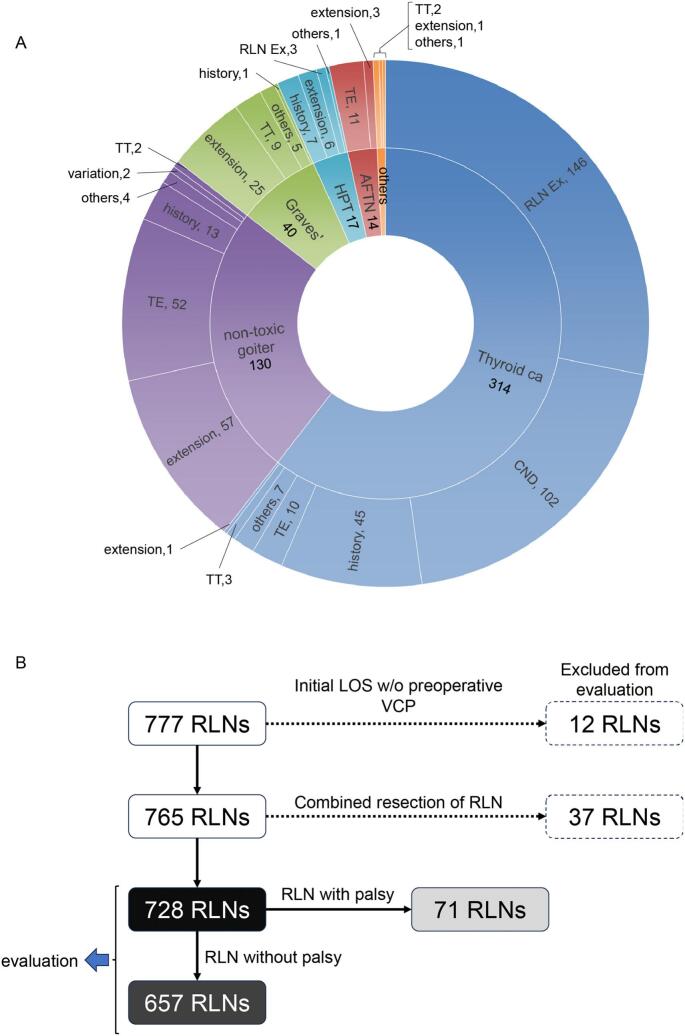
(A) Main reasons for application of I-IONM in 510 patients. Nine patients have multiple diseases and I-IONM was conducted for multiple reasons in these patients. HPT, hyperparathyroidism including parathyroid cancer; AFTN, autonomously functioning thyroid nodule(s); RLN Ex, suspicious of involvement of recurrent laryngeal nerve; CND, central neck lymph node dissection; history, previous history of surgery including paratracheal area or external beam radiation therapy to neck; TE, total endoscopic thyroidectomy; TT, total thyroidectomy; extension, substernal extension of goiter/tumor or large goiter/tumor; variation, anatomical variation (*e.g*. aberrant right subclavian artery). (B) Among 777 RLNs, 49 RLNs were excluded because of initial loss of signal (LOS) even after both tube reposition under direct laryngoscopy and administration of a reversal agent, regardless of intact preoperative vocal cord movement and no RLN involvement observed during surgery (12 RLNs), or combined resection of RLN for invasion of cancer (37 RLNs). The remaining 728 RLNs were evaluated. Among them, postoperative vocal cord paralysis (VCP) was observed in 71 RLNs.

I-IONM was conducted in 777 RLNs at risk of 510 patients for 519 diseases which is 28.6 % of total 1784 patients during in the period of this study. The median age at surgery was 56 years (range: 7–95 years). Females and males comprise 382 (74.9 %) and 128 (25.1 %), respectively. The 519 diseases consist of 314 thyroid cancer, 130 non-toxic goiter, 40 Graves' disease, 17 primary hyperparathyroidism (including parathyroid cancer), 40 autonomously functioning thyroid nodule(s) (AFTN), and 4 other diseases (Fig. 1A). The median observation period is 1237 days (range: 46–3174 days). The main reasons for IONM application are as follows: in thyroid cancer, suspicious of RLN involvement (146 patients), central neck dissection (102), followed by previous surgical history of paratracheal area including reoperation for recurrence(s) or previous external beam radiation therapy to neck (45), total endoscopic thyroidectomy (10), and others; in non-toxic goiter, substernal extension or large goiter (57), total endoscopic thyroidectomy (52), followed by previous history of surgery or external radiation therapy including paratracheal area (13), and others; in Graves' disease, large goiter (25) and others; in primary hyperparathyroidism, previous history of surgery or external radiation therapy including paratracheal area (7), substernal extension (6), followed by suspicious of RLN involvement (3) and others; in AFTN, total endoscopic thyroidectomy (11), followed by substernal extension (3) and others were main reason for IONM, respectively.

Among 777 RLNs, 12 RLNs (1.54 %) were excluded because of initial LOS below noise even after both tube reposition under direct laryngoscopy and administration of a reversal agent, regardless of preoperative intact vocal cord mobility and intrathyroidal disease without RLN involvement that was confirmed during surgery, 37 RLNs were excluded because of combined resection of RLN for invasion of cancer (Fig. 1B). The remaining 728 RLNs were evaluated in this study. Among them, postoperative VCP (RLN palsy) was observed in 71 RLNs (9.8 %), that is acceptable considering that a quantitative I-IONM was performed preferentially in the high risk patients in this study. Postoperative VCP with type I and type II LOS was observed in 49 (69.0 %) and 14 (19.7 %) RLNs at risk, respectively. Postoperative VCP without LOS throughout operation was seen in 8 (11.3 %) RLNs. For these 728 RLNs, the median follow-up period was 1322 days (range: 46–3174 days). Quantitative V2 and R2 amplitudes were obtained in 642 and 693 RLNs at risk, respectively.

### A quantitative V2 amplitude excellently predicts postoperative vocal cord paralysis

**Fig. 2:**
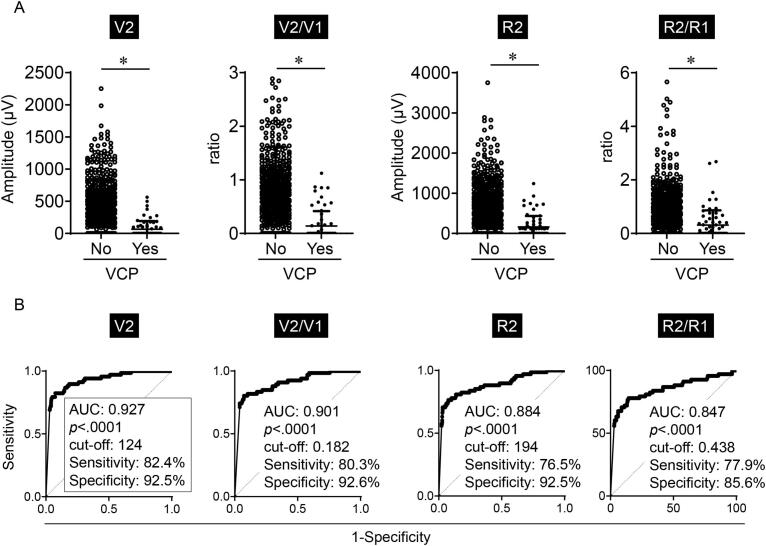
A quantitative V2 amplitude excellently predicts postoperative VCP. (A) Scatter plots of quantitative amplitudes of V2 and R2, and ratios of V2 to V1 (V2/V1), and R2 to R1 (R2/R1), considering postoperative VCP. *p < .0001. Bar, mean ± standard deviation (SD). (B) ROC curves of quantitative amplitudes (V2 and R2), and amplitude ratios (V2/V1 and R2/R1) on postoperative VCP. V2 amplitude most excellently predicts VCP. AUC, area under curve.

We first evaluated the actual quantitative amplitudes obtained by I-IONM considering postoperative VCP as shown in Fig. 2A. As expected, an individual amplitude decrease represented by lower V2 to V1 ratio or lower R2 to R1 ratio significantly reflect VCP (*p* < .0001). Quantitative amplitudes of V2 (66.2 ± 39.4 μV) and R2 (162.0 ± 59.1 μV) in cases with postoperative VCP are significantly lower than V2 (487.4 ± 13.6 μV) and R2 (727.6 ± 19.5 μV) in the counterparts (p < .0001), and dramatically reflect VCP.

**Fig. 3:**
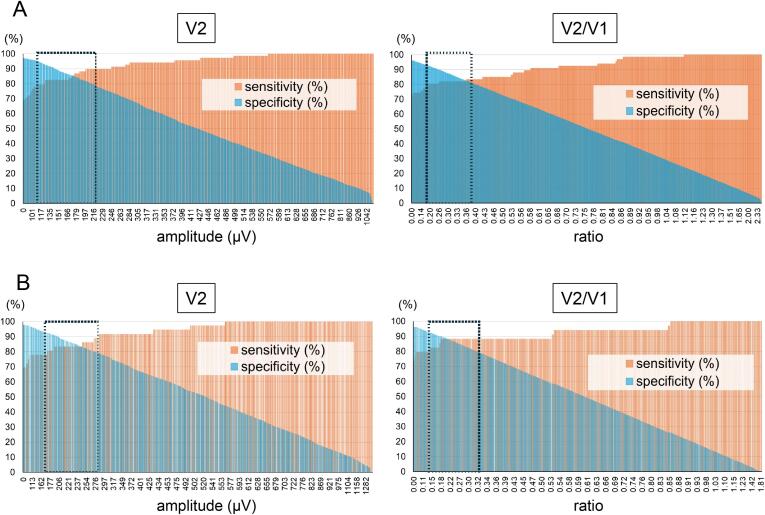
Bar graphs of sensitivity and specificity of V2 amplitude or V2/V1 ratio on predicting VCP. (A) whole cases. (B) cases with initial V1 ≥ 500 μV. Specificities show linear decline in inverse proportion to both V2 amplitude and V2/V1 ratio. A square dotted line indicates cases that satisfy both sensitivity and specificity ≥80 %.

A cut-off determined by an ROC curve reflects postoperative VCP excellently in all V2, R2, V2 to V1 ratio (V2/V1), and R2 to R1 ratio (R2/R1) with very high sensitivity and specificity (Fig. 2B). A negative predictive value for VCP is excellently high in all V2, R2, V2/V1, and R2/R1 (Supplementary Table 1). However, a positive predictive value for VCP of R2/R1 (0.371) is low compared to that of V2 (0.566), R2 (0.525), and V2/V1 (0.564). Interestingly, the AUC of V2 was the highest and a cut-off 124 μV of V2 most excellently predicts postoperative VCP with the highest levels of sensitivity (82.4 %), specificity (92.5 %), and both positive (0.566) and negative (0.978) predictive values (Fig. 2B and Supplementary Table 1). Although 124 μV of V2 was the cut-off value according to the ROC curve, the wide range of V2 amplitudes (117 to 216 μV) can predict postoperative VCP with both high sensitivity (>80 %) and specificity (>80 %) (Fig. 3A). Additionally, an optimal cut-off of V2/V1 0.182 was significantly lower than 0.5 that has been generally applied [9]. These results suggest that a quantitative V2 amplitude predicts postoperative VCP among patients at least comparable to an amplitude decrease in each individual patient.

**Fig. 4:**
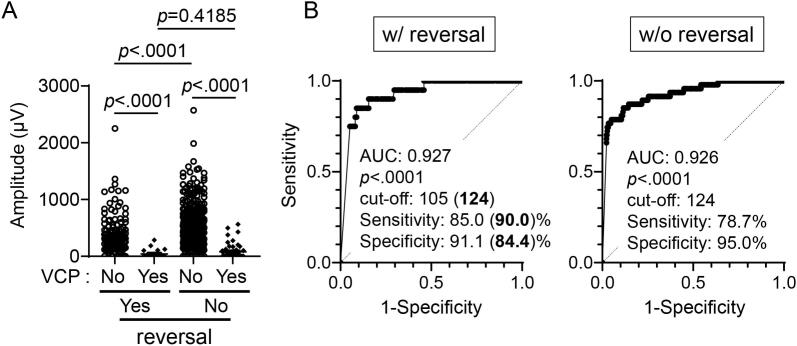
Quantitative V2 amplitudes on postoperative VCP considering reversal agent (sugamadex) use. (A) Scatter plots of quantitative amplitudes of V2 on VCP considering reversal agent administration. Bar, mean ± SD. (B) ROC curves of V2 amplitudes on postoperative VCP. A cut-off 124 μV of V2 excellently predicted postoperative VCP regardless of whether or not reversal agent was used. The sensitivity and specificity in the parentheses represent those at a cut-off 124 μV.

Since an amplitude obtained by I-IONM may be affected by a neuromuscular blocking agent on anesthetic induction, quantitative V2 amplitudes were separately evaluated considering use of a reversal agent, sugamadex. In this study, sugamadex was administered for a patient with a relatively low initial amplitude of either V1 or R1, which was <200 μV (116 patients (22.7 %)/155 RLNs (21.3 %)). The V2 amplitudes of cases without VCP with sugamadex (381.3 ± 28.1 μV) were still significantly lower than that of the counterparts without sugamadex (519.9 ± 16.1 μV) (Fig. 4A). These results suggest that other factors of patient side or IONM set-up may affect lower amplitudes in this group. On the other hand, in the cases with postoperative VCP, no statistical difference was seen in V2 amplitudes by sugamadex use. Nevertheless, V2 amplitudes of the cases without VCP were still significantly higher than that of the counterparts with VCP in the cases both with and without sugamadex administration (*p* < .0001). Moreover, a cut-off 124 μV of V2 excellently predicts postoperative VCP with high sensitivity and specificity in the cases both with and without sugamadex as well (Fig. 4B). Collectively, quantitative V2 amplitudes can be evaluated together regardless of sugamadex administration as long as quantitative V2 amplitudes are sufficient.

**Fig. 5:**
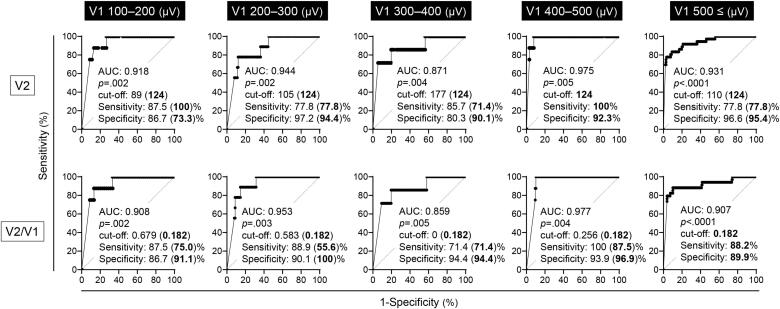
ROC curves of V2 amplitudes (top lane) and V2/V1 (bottom lane) in each range of initial V1 amplitudes. A cut-off 124 μV of V2 excellently predicts postoperative VCP at an initial V1 amplitude ≥100 μV. The sensitivity and specificity in the parentheses represent those at a cut-off 124 μV of V2, or those at a cut of 0.182 of V2/V1.

We next evaluated V2 amplitudes separately according to ranges of initial V1 amplitude to know a sufficient amplitude level of initial V1 for predicting postoperative VCP. Surprisingly, a cut-off 124 μV of V2 can be applied to predict postoperative VCP in all ranges of initial V1 amplitudes equal or >100 μV with high sensitivities and specificities (Fig. 5). A cut-off 124 μV of V2 is comparable to a cut-off 0.182 of V2/V1 ratio in each range. In cases limited with initial V1 ≥ 500 μV (*i.e.* guideline recommendation), the sensitivity and specificity for VCP of a V2 cut-off 124 μV was 77.8 % and 95.4 %, respectively. Although the specificity was higher than that of a V2/V1 cut-off 0.182 (89.9 %), the sensitivity was lower than that of a V2/V1 cut-off 0.182 (88.2 %). The range of V2 amplitudes which fulfill both high sensitivity (>80 %) and high specificity (>80 %) was 170 to 279 μV in cases with V1 ≥ 500 μV (Fig. 3B).

### Association of quantitative V2 amplitudes with clinical factors

**Fig. 6:**
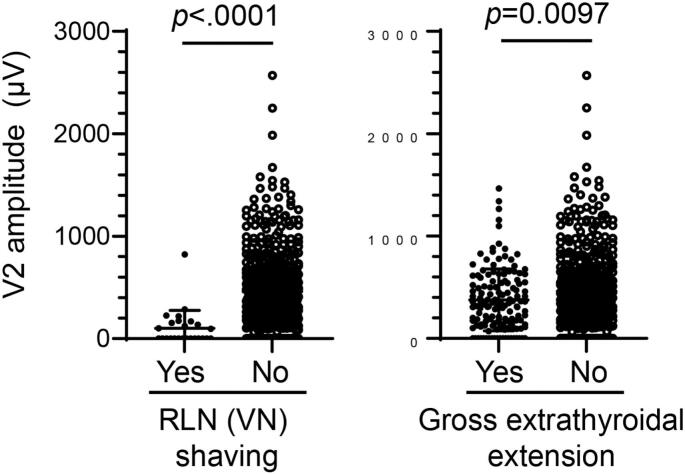
Quantitative V2 amplitudes are significantly lower in cases with RLN (or vagus nerve [VN]) shaving or gross extrathyroidal extension than that of the counterparts. Scatter plots of quantitative V2 amplitudes taking LN (or VN) shaving (left) or gross extrathyroidal extension (right) into account. Bar, mean ± SD.

In correlation analyses of V2 amplitudes at a cut-off 124 μV with clinical factors (Table 1), RLN or vagus nerve (VN) shaving procedure for involvement of cancer and gross extrathyroidal extension of cancer are significantly correlated with low V2 amplitudes. Indeed, quantitative V2 amplitudes in cases with RLN (or VN) shaving (100.2 ± 35.2 μV) are significantly lower than that of the counterparts (457.4 ± 14.0 μV) (Fig. 6 left). Similarly, quantitative V2 amplitudes in cases with gross extrathyroidal extension (375.8 ± 25.7 μV) are significantly lower than that of the counterparts (462.2 ± 16.0 μV) (Fig. 6 right). On the other hand, no correlation was observed in large goiter or mass, mediastinal extension, previous history of radiation therapy or surgery including paratracheal area, or central neck dissection. These results may be because RLN shaving and gross extrathyroidal extension are inevitable factors regardless of careful manipulation during surgical procedure for preserving RLN integrity.

**Table 1:** Correlation analysis of clinical variables with V2 amplitudes in patients without RLN resection.

Variables	No. of RLNs (*n* = 642)	V2 (μV)				p^a^
		≤124		>124		
		n	%	n	%	
Gender						0.247
Male	168	30	17.9	138	82.1	
Female	474	68	14.3	406	85.7	
Age						0.279
≤55	314	43	13.7	271	86.3	
>55	328	55	16.8	273	83.2	
Malignancy of disease						0.538
Benign	180	30	16.7	150	83.3	
Malignant	462	68	14.7	394	85.3	
Large goiter or mass^b^						0.809
Yes	70	10	14.3	60	85.7	
No	572	88	15.4	484	84.6	
Mediastinal extension						0.263
Yes	65	13	20.0	52	80.0	
No	577	85	14.7	492	85.3	
Gross extrathyroidal extension						0.004
Yes	139	32	23.0	107	77.0	
No	503	66	13.1	437	86.9	
Past history of radiation therapy^c^ to neck						0.176
Yes	10	0	0.0	10	0.0	
No	632	98	15.5	534	84.5	
Past surgical history of paratracheal area						0.961
Yes	73	11	15.1	62	84.9	
No	569	87	15.3	482	84.7	
RLN (or VN) shaving on a side of RLN at risk						<0.0001
Yes	25	17	68.0	8	32.0	
No	617	81	13.1	536	86.9	
Central neck lymph node dissection						0.155
Yes	414	57	13.8	357	86.2	
No	228	41	18.0	187	82.0	

Abbreviations: RLN, recurrent laryngeal nerve; VN, vagus nerve.

a A chi-square test or a Fischer's exact test.

b Large goiter >100 g or mass >6 cm.

c Radiation therapy includes radioactive iodine therapy (RAI) and external beam radiotherapy.

### Recovery from vocal cord paralysis and quantitative amplitudes obtained by I-IONM

**Fig. 7:**
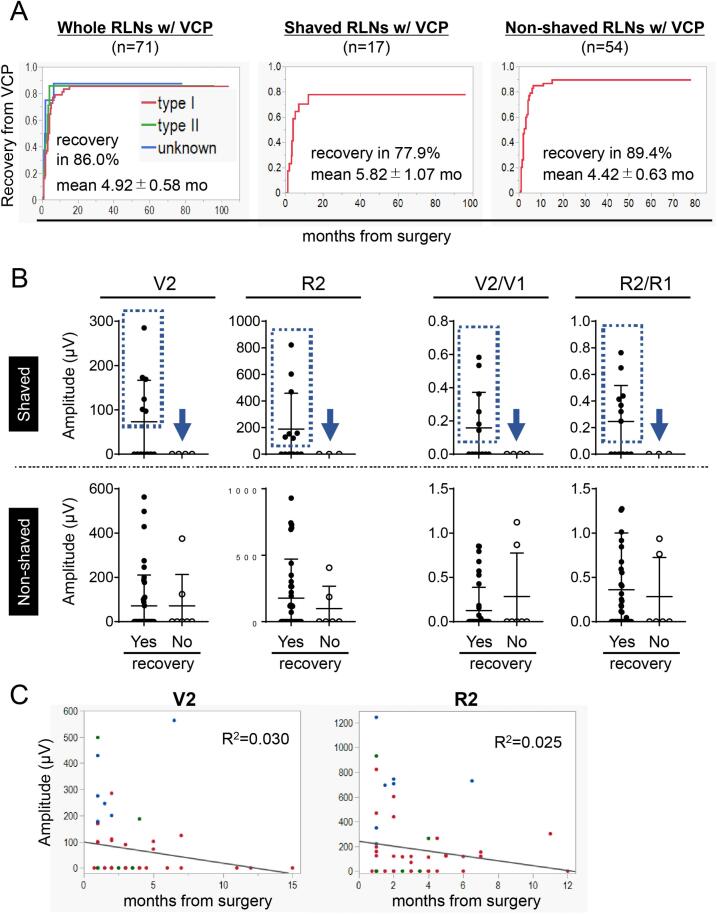
Evaluation of quantitative amplitudes and recovery from VCP in patients with postoperative VCP (71 RLNs). (A) Cumulative rates of recovery from VCP in whole RLNs (respective curve represents type I, II, or unknown type VCP as shown) (*n* = 71, left), RLNs with shaving (*n* = 17, middle), and RLNs without shaving (*n* = 54, right). mo, months. (B) Scatter plots of V2, R2, V2/V1, and R2/R1 considering recovery from VCP. RLNs with shaving (top lane), RLN without shaving (bottom lane). In cases with shaved RLNs, positive amplitudes of V2 or R2 are only observed in cases with recovery from VCP. (C) Correlation of quantitative amplitudes of V2 or R2 and time to recovery from VCP. Each color of plot indicates type of VCP (type I: red; type II: green; unknown: blue). (For interpretation of the references to color in this figure legend, the reader is referred to the web version of this article.)

Next, we focused on recovery from VCP. Postoperative VCP was observed in 71 RLNs at risk which comprise 9.8 % of this cohort. RLN shaving was performed for involvement by cancer in 17 RLNs. Recovery from VCP was observed in 86.0 % and average period to recovery from VCP are 4.92 ± 0.58 months in whole RLNs (Fig. 7A). There was no difference in recovery rate among types of VCP: 87.2 (type I), 85.7 (type II), and 87.5 % (unknown type). Average period to recovery from VCP was shorter in type II (2.61 ± 0.32 months) and unknown type (2.69 ± 0.85 months) than that in type I (5.36 ± 0.71 months). Recovery from VCP in RLN-shaved cases (77.9 %) were less than that in RLN non-shaved cases (89.4 %), and the average period to recovery from VCP were longer in shaved cases (5.82 ± 1.07 months) than that in non-shaved cases (4.42 ± 0.63 months).

In the RLN-shaved cases, a positive V2 or R2 amplitude was only observed in cases with recovery, and predicted recovery from VCP although recovery was also seen in cases with a negative V2 or R2 amplitude as well (Fig. 7B, top). Of note, in the non-shaved cases, there was no difference in V2 or R2 amplitudes on recovery from VCP (Fig. 7B, bottom). In terms of relationship between time to recovery and V2 or R2 amplitudes in 57 recovered RLNs, there was no statistical relationship between a quantitative amplitude and time to recovery in this study. However, scatter plots of amplitudes deem to decline according to time, further study is needed to accumulate case volume.

**Fig. 8:**
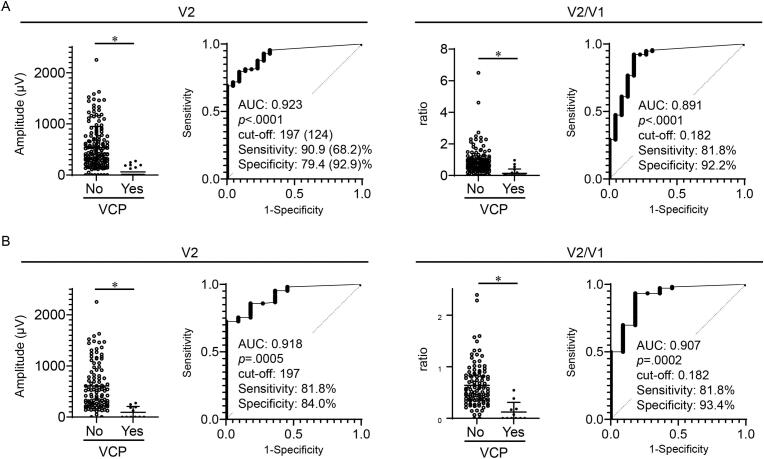
Evaluation of V2 quantitative amplitudes and V2/V1 ratio on postoperative VCP in the validation set. Scatter plots and ROC curves of V2 quantitative amplitudes and V2/V1 ratios on VCP in the whole validation cases (A, 177 RLNs at risk) or cases with V1 ≥ 500 μV (B, 117 RLNs at risk). In the validation set, 197 μV was the optimal cut-off V2 amplitude for predicting VCP with high sensitivity and specificity in both whole cases and cases with initial V1 ≥ 500 μV. *p < .0001. Bar, mean ± SD.

### A quantitative V2 amplitude also predicted postoperative VCP in an independent validation cohort

To validate usefulness of a quantitative V2 amplitude as a second option for VCP prediction, an independent cohort of 144 patients (177 RLNs at risk) were evaluated. Supplementary Table 2 shows patient characteristics. Patients who underwent combined resection of RLN or VN were not included in this cohort. Seven RLNs at risk were shaved for cancer involvement. Similarly, the range of V2 amplitudes (148 to 205 μV) predicted VCP with both high sensitivity (≥80 %) and specificity (≥80 %). The most optimal cut-off amplitude was increased to 197 μV (Fig. 8A). In cases with V1 ≥ 500 μV (117 RLNs at risk), the range of V2 amplitudes (194 to 225 μV) predicted VCP with both high sensitivity (≥80 %) and specificity (≥80 %). The V2 cut-off 197 μV also showed high sensitivity (81.8 %) and high specificity (84.0 %) (Fig. 8B). The V2 amplitude range 194 to 205 μV fulfill both high sensitivity (≥80 %) and specificity (≥80 %) in the study set and the validation set, implying that the V2 amplitude ranges are useful to predict VCP especially in cases where initial exposure of vagus nerve is difficult.

## Discussion

Intraoperative neuromonitoring (IONM) during thyroid and parathyroid surgery has been widely accepted as the gold standard of nerve identification [[1], [2], [3]]. A remarkable decrease or loss of the electromyogram amplitude of IONM reflects RLN palsy and VCP. However, because of wide range variation of individual amplitudes obtained by IONM, clinical significance of actual quantitative amplitudes has been elusive. In the present study, we evaluated a usefulness of a quantitative amplitude *per se* obtained by IONM, and assessed the potential for universal application among individual patients as a second option. We found that a quantitative amplitude of vagus nerve at the end of surgery (V2) can excellently predict postoperative VCP universally among patients at an optimal range of cut-offs. Considering the optimal range of V2 amplitudes on VCP prediction in the study cohort (117 to 216 μV) and the validation cohort (194 to 225 μV), V2 ≥ 200 μV would be best to use as a universal cut-off. Interestingly, the predictive impact on postoperative VCP is even stronger than an amplitude decrease in each individual patient. To our knowledge, this study is the first report that focusing on universal utilities of quantitative IONM amplitudes *per se*, and the results may be especially useful in some circumstances with unavoidable insufficient initial exposure of vagus nerve because of tumor extension, adhesive operation field and so forth.

A optimal range (117 to 216 μV) and a cut-off 124 μV of quantitative V2 amplitude could excellently predict VCP with high sensitivity and specificity at broad range of an initial V1 amplitude ≥100 μV as shown by separate analyses according to an initial V1 amplitude in this study. Surgeons generally tend to try to obtain IONM amplitude as much as possible in order to make sure V1 ≥ 500 μV and RLN integrity during surgical procedure, supposedly results in remarkable variation among individual patients. In addition, excessive struggling to expose nerves may cause unnecessary trauma to tissues. Our results suggest that V2 ≥ 200 μV would be enough to predict postoperative RLN integrity (Fig. 5, Fig. 8), implying that it is not necessary to stick to gain V2 amplitude such as ≥500 μV. Therefore, the advantages of a universal V2 application are that clear interpretation of an obtained V2 amplitude and potential to simplification of IONM procedure, leading to decrease of surgeon's stress or minimization of surgical manipulation particularly in cases with unavoidable insufficient initial exposure of vagus nerve.

An amplitude decrease of RLN stimulation represented by R2/R1 showed lower positive predictive value (Supplementary Table 1). Although the operators in this study tried to stimulate most proximal part of exposed RLN at the end of surgery, traction during surgical procedure can still affect global RLN integrity. Great variations of amplitude reduction caused by a tensile stress were reported among individuals in a porcine model [10]. These results also support the advantage of V2 amplitudes to predict postoperative RLN integrity.

In this study, a reversal agent sugamadex was administered only for a patient with a relatively low initial amplitude of either V1 or R1 < 200 μV (22.7 % of patients, 21.3 % of RLNs) in order to avoid its hypersensitivity reactions and anaphylaxis [11,12]. The V2 amplitudes of cases without postoperative VCP with sugamadex were still significantly lower than that of the counterparts without sugamadex although a cut-off 124 μV of V2 excellently predicts postoperative VCP regardless of sugamadex administration in this study. These results suggest that other factors such as individual variations of nerve sensitivity or technical IONM set-up reasons may affect lower amplitudes in this group in addition to residual effect of a neuromuscular relaxant rocuronium. The technique of Train of Four (TOF) monitoring may be able to exclude a neuromuscular relaxant factor by the residual effect of rocuronium [13,14].

The limitations of this report: this study is retrospectively conducted in a single institution and the patients in this cohort may have undergone unified and standardized operations performed by limited surgeons; significant number of patients had lower baseline V1 amplitude than 500 μV which is recommended by the INMSG guideline [2,3] although V2 amplitude still predicted postoperative VCP comparably even in the limited cases with V2 ≥ 500 μV; the patient cohort in this study is a heterogenous group consisting of various diseases. We think that this heterogeneity is also strength of this study reflecting real-world clinical practice. Further validation among multiple surgeons in multiple institutions is warranted.

In conclusion, we report that a quantitative amplitude of vagus nerve at the end of surgery can universally predict VCP with an optimal range of cut-offs comparably to individual amplitude decrease. The results in this study may give rationale to make easier and simplify the interpretation of a quantitative amplitude of IONM as a second option.

The following are the supplementary data related to this article.

Supplementary Table 1 PPV and NPV for VCP. Supplementary Table 1

Supplementary Table 2 Characteristics of RLNs at risk. Supplementary Table 2

## CRediT authorship contribution statement

**Hiroshi Katoh:** Conceptualization, Data curation, Formal analysis, Investigation, Methodology, Project administration, Supervision, Validation, Visualization, Writing – original draft, Writing – review & editing. **Riku Okamoto:** Data curation, Formal analysis. **Kanako Naito:** Data curation. **Tomoya Mitsuma:** Data curation. **Mariko Kikuchi:** Data curation. **Takaaki Tokito:** Data curation. **Takeshi Naitoh:** Supervision, Writing – review & editing. **Naoki Hiki:** Supervision, Writing – review & editing. **Yusuke Kumamoto:** Supervision, Writing – review & editing. **Takafumi Sangai:** Supervision, Writing – review & editing.

## Ethics approval

This study was approved by the ethics committee (IRB) of the Kitasato University School of Medicine (#B22-004), and was performed in accordance with the clinical research guidelines of the IRB of the Kitasato University School of Medicine. All individuals gave written informed consent for pathologic assessment and routine blood sample analyses on their samples, and clinical data. The study was conducted in accordance with the Declaration of Helsinki (as revised in 2013).

## Precis

A quantitative amplitude of vagus nerve after surgical procedure obtained by intraoperative nerve monitoring (IONM) can predict vocal cord paralysis universally among patients in thyroid/parathyroid surgery as a simple and a second optional method.

## Funding

None.

## Declaration of competing interest

The authors declare that they have no known competing financial interests or personal relationships that could have appeared to influence the work reported in this paper.
